# Automatic CNN-based detection of cardiac MR motion artefacts using k-space data augmentation and curriculum learning

**DOI:** 10.1016/j.media.2019.04.009

**Published:** 2019-04-22

**Authors:** Ilkay Oksuz, Bram Ruijsink, Esther Puyol-Antón, James R. Clough, Gastao Cruz, Aurelien Bustina, Claudia Prietoa, Rene Botnar, Daniel Rueckert, Julia A. Schnabel, Andrew P. King

**Affiliations:** a School of Biomedical Engineering & Imaging Sciences, King’s College, London, UK; b Guy’s and St Thomas’ Hospital NHS Foundation Trust, London, UK; c Biomedical Image Analysis Group, Imperial College, London, UK

**Keywords:** Cardiac MR motion artefacts, Image quality assessment, Artifact, Convolutional neural networks, LSTM

## Abstract

Good quality of medical images is a prerequisite for the success of subsequent image analysis pipelines. Quality assessment of medical images is therefore an essential activity and for large population studies such as the UK Biobank (UKBB), manual identification of artefacts such as those caused by unanticipated motion is tedious and time-consuming. Therefore, there is an urgent need for automatic image quality assessment techniques. In this paper, we propose a method to automatically detect the presence of motion-related artefacts in cardiac magnetic resonance (CMR) cine images. We compare two deep learning architectures to classify poor quality CMR images: 1) 3D spatio-temporal Convolutional Neural Networks (3D-CNN), 2) Long-term Recurrent Convolutional Network (LRCN). Though in real clinical setup motion artefacts are common, high-quality imaging of UKBB, which comprises cross-sectional population data of volunteers who do not necessarily have health problems creates a highly imbalanced classification problem. Due to the high number of good quality images compared to the relatively low number of images with motion artefacts, we propose a novel data augmentation scheme based on synthetic artefact creation in k-space. We also investigate a learning approach using a predetermined curriculum based on synthetic artefact severity. We evaluate our pipeline on a subset of the UK Biobank data set consisting of 3510 CMR images. The LRCN architecture outperformed the 3D-CNN architecture and was able to detect 2D+time short axis images with motion artefacts in less than 1ms with high recall. We compare our approach to a range of state-of-the-art quality assessment methods. The novel data augmentation and curriculum learning approaches both improved classification performance achieving overall area under the ROC curve of 0.89.

## 1 Introduction

With developments in image acquisition schemes and machine learning algorithms, medical image analysis techniques are taking on increasingly important roles in clinical decision making. An important and often overlooked step in automated image analysis pipelines is the assurance of image quality - high accuracy requires good quality medical images. Cine cardiac magnetic resonance (CMR) images are instrumental in assessment of cardiac health, deriving metrics of cardiac function (e.g. volumes and ejection fractions), and investigating myocardial wall motion abnormalities. The CMR is often acquired for patients, who already have existent cardiac diseases, more likely to have arrythmias, have difficulties with breath-holding or remaining still during acquisition. Therefore, the images can contain a range of image artefacts (Ferreira et al., 2013), and assessing the quality of images acquired by MR scanners is a challenging problem. Misleading conclusions can be drawn when the original data are of poor quality. Traditionally, images are visually inspected by one or more experts, and those showing an insufficient level of quality are excluded from further analysis. However, visual assessment is time consuming and prone to variability due to inter-rater and intra-rater variability.

**Fig. 1:**
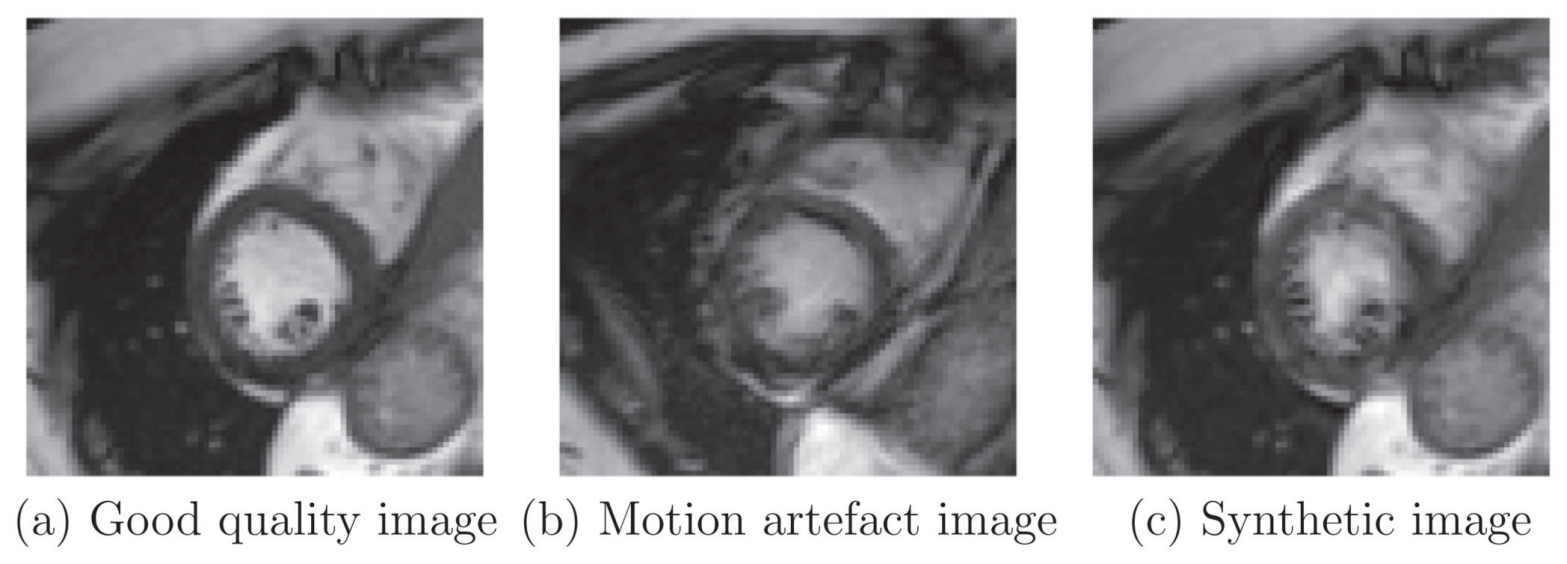
Examples of a good quality cine CMR image (a), an image with blurring motion artefacts (b), and a k-space corrupted image (c). The k-space corruption process is able to simulate realistic motion-related artefacts. (Please see videos in supplementary material.).

The UK Biobank is a large-scale study with all data accessible to researchers worldwide. The CMR images in UK Biobank will eventually consist of 100,000 subjects (Petersen et al., 2015). To maximise the research value of this and other similar data sets, automatic quality assessment tools are essential. One specific challenge in CMR is motion-related artefacts such as mistriggering, arrhythmia and breathing artefacts. These can result in temporal and/or spatial blurring of the images, which makes subsequent processing difficult (Ferreira et al., 2013). These type of artefacts are more common in real clinical acquisitions, and there would be great value for motion artefact detection mechanisms being deployed in the MR scanner. For example, these types of artefact can lead to erroneous quantification of myocardial wall motion, which is an important indicator in cardiac functional assessment. Examples of a good quality image and an image with blurring motion artefacts are shown in Fig. 1a-b for a short-axis view cine CMR scan.

In this paper, we propose a deep learning based approach for fully automated motion artefact detection in cine CMR short axis images. A novel data augmentation strategy based on synthetic artefact creation in k-space and a curriculum learning scheme based on the synthetic artefacts with different levels of severity (Fig. 8) is also proposed. An analysis of multiple deep learning architectures and learning mechanisms is also presented. This paper builds upon our previously presented work (Oksuz et al., 2018), in which we proposed the use of synthetically generated mistriggering artefacts in training a Convolutional Neural Network (CNN). Here, we extend this idea to include both breathing and mistriggering artefacts and also use different levels of corruption in order to produce a curriculum of realistic artefact images of varying severity (Fig. 1c) to improve training.

The remainder of this paper is organised as follows. In Section 2, we first present an overview of the relevant literature in image quality assessment and the data imbalance problem, which our novel extensions are based on. Then, we review the literature on curriculum learning, and present our novel contributions in this context. In Section 3, we provide details of the clinical data sets used. In Section 4 we describe the deep learning models that we have utilised for classification, including descriptions of the novel data augmentation and curriculum learning approaches. Results are presented in Section 5, while Section 6 discusses the findings of this paper in the context of the literature and proposes potential future work directions.

## 2 Related works

In this section, we provide an overview of the relevant literature on image quality assessment, data imbalance and curriculum learning with a focus on applications in medical image analysis.

### 2.1 Image quality assessment

An automatic image quality assessment (IQA) algorithm, given an input image, tries to predict its perceptual quality. The perceptual quality of an image is usually defined as the mean of the individual ratings of perceived quality assigned by human observers. Early works on IQA focused on using Natural Scene Statistics (NSS) to predict the naturalness of the images. For example, Mittal et al. (2013) proposed the Naturalness Image Quality Evaluator (NIQE) model, which constructed a collection of statistical features based on a space domain NSS model. Moorthy and Bovik (2011) proposed a two-stage framework for estimating quality based on NSS models, involving identification- and distortion-specific quality assessment. More recently, Convolutional Neural Networks (CNNs) have been utilised for image quality assessment Kang et al. (2014) and Talebi and Milanfar (2018) proposed a novel loss function definition and focused on the distribution of the ground truth quality scores.

IQA is an essential step for analysing large medical image data sets (see Chow and Paramesran, 2016 for a comprehensive review). Early efforts in medical imaging focused on quantifying the image quality of brain MR images. Woodard and Carley-Spencer (2006) defined a set of 239 no-reference (i.e. without the need for ground truth image) image-quality metrics (IQMs). However, the IQMs were calculated on image pairs with simplistic distortions such as Gaussian noise or intensity nonuniformity, which are unlikely to adequately capture the nature of real world MR image artefacts. Mortamet et al. (2009) proposed two IQMs focused on detecting artefacts in the air region surrounding the head. They applied these IQMs in 749 scans from the Alzheimers Disease Neuroimaging Initiative (ADNI) database. However, many potential sources of uncontrolled variability exist between studies and sites, including MR protocols, scanning settings, participant instructions, inclusion criteria, etc. The thresholds they proposed on their IQMs are unlikely to generalise beyond the ADNI database.

Trends in the computer vision literature have heavily influenced medical image quality assessment techniques. CNNs have been utilised for image quality assessment for compressed images in the computer vision literature with considerable success (Kang et al., 2014). This success has motivated the medical image analysis community to utilise them on multiple image quality assessment challenges such as fetal ultrasound (Wu et al., 2017) and echocardiography (Abdi et al., 2017a). These two techniques use 2D images and assess quality using pre-trained neural networks. A more recent study (Abdi et al., 2017b) aimed to utilise temporal information using a Long Short Term Memory (LSTM) architecture to improve the accuracy of image quality assessment. Küstner et al. (2018a) utilised a patch-based CNN architecture to detect motion artefacts in head and abdomen MR scans to achieve spatially-aware probability maps. In more recent work, Küstner et al. (2018b) proposed to utilise a variety of features and train a deep neural network for artefact detection. The authors made use of an active learning strategy to detect low quality images due to the lack of sufficient training data.

In the context of CMR, the literature has mostly focused on missing apical and basal slice detection (Zhang et al., 2016). Missing slices adversely affect the accurate calculation of the left ventricular volume and hence the derivation of cardiac metrics such as ejection fraction. Another study (Zhang et al., 2017) used Generative Adversarial Networks in a semi-supervised setting to improve the performance of missing slice detection. Tarroni et al. (2018) proposed to use a decision forest approach for heart coverage estimation, inter-slice motion detection and image contrast estimation in the cardiac region. CMR image quality has also been linked with automatic quality control of image segmentation in Robinson et al. (2017). Lorch et al. (2017) investigated synthetic motion artefacts and used histogram, box, line and texture features to train a random forest algorithm to detect different artefact levels. However, their algorithm was tested only on artificially corrupted synthetic data and aimed only at detecting breathing artefacts.

### 2.2 Data imbalance

Data imbalance is a significant factor that influences the stability of machine learning algorithms (Chawla, 2010). The fundamental issue with the imbalanced learning problem is the ability of imbalanced data to significantly compromise the performance of most standard learning algorithms. This occurs because the skewed distribution of class instances can lead the classification algorithms to be biased towards the majority class in classification tasks. Therefore, the features relevant to the minority class are not learned adequately. As a result, standard classifiers (classifiers that do not consider data imbalance) tend to misclassify the minority samples into majority samples, which results in poor classification performance (Wang et al., 2016). How to deal with imbalanced data sets is a key issue in classification and it has been well explored over past decades. Until now, this issue has been solved mainly in two ways: sampling techniques and cost sensitive methods.

#### 2.2.1 Sampling techniques

Sampling techniques aim to address the data imbalance problem by generating a balanced data set by sampling the full data set (Estabrooks et al., 2004). Random over-sampling is one of the simplest sampling methods. It randomly duplicates a certain number of samples from the minority class and then augments them into the original data set (Han et al., 2005). Conversely under-sampling randomly removes a certain number of instances from the majority class to achieve a balanced data set. In general, random over-sampling may lead to overfitting while random under-sampling may result in insufficient training data.

#### 2.2.2 Cost sensitive learning

In addition to sampling techniques, another way to deal with the data imbalance problem is cost sensitive learning. In contrast to sampling methods, cost sensitive learning methods solve the data imbalance problem by assigning different costs to mis-classifying majority and minority samples (Khan et al., 2018). An objective function for cost sensitive learning can be constructed based on the aggregation of the overall cost on the whole training set. Although cost sensitive algorithms can significantly improve classification performance, they are only applicable when the specific costs of misclassification are known. Unfortunately, in many applications a cost with appropriate weights is hard to define (Maloof, 2003).

#### 2.2.3 Data imbalance problem for neural networks

In the area of neural networks, many efforts have been made to address the data imbalance problem. Nearly all of the work falls into one of the main streams mentioned above. Zhou and Liu (2006) empirically studied the effect of sampling and threshold-moving in training cost sensitive neural networks. Both over-sampling and under-sampling techniques were used to modify the distribution of the training data set. To avoid the potential issues with these basic approaches, a more complex sampling method was proposed. The synthetic minority over-sampling technique (SMOTE) has proven to be quite powerful and has achieved a great deal of success in various applications (Han et al., 2005). SMOTE creates artificial data based on the similarities between existing minority samples. Our approach in this paper is related to the SMOTE approach in that we propose to generate synthetic data for the minority class using prior knowledge of the process of cine MR image acquisition.

### 2.3 Curriculum learning

A curriculum determines a sequence of training samples, which essentially corresponds to a list of samples ranked in ascending order of learning difficulty. In a pioneering work Elman (1993) studied the effect of a learning structure on a synthetic grammar task. His work was inspired by language learning in children and demonstrated that a neural network was able to learn the grammar when training data was presented from simple to complex order and failed to do so when the order was random.

The idea of learning easy things first has been an active research topic in computer vision (Lee and Grauman, 2011). Bengio et al. (2009) demonstrated that curriculum learning resulted in better generalisation and faster learning on synthetic vision and word representation learning tasks. Pentina et al. (2015) investigated the effect of curriculum learning in a multi-task learning setup and proposed a model to learn the order of multiple tasks. They illustrated the superiority of learning tasks sequentially instead of learning tasks jointly. Avramova (2015) applied curriculum learning to a natural image classification task by training a CNN from scratch. Weinshall et al. (2018) investigated the robustness of curriculum learning in common computer vision image classification tasks and highlighted the superiority in convergence. Gui et al. (2017) proposed a curriculum learning strategy on facial expression classification, where they order the training samples according to their difficulty to classify them. The authors have illustrated improved accuracy at emotion classification using curriculum learning training.

Recently, the idea of curriculum learning has been utilised for medical imaging challenges. Jesson et al. (2017) proposed to use patches of different complexity to train a network for lung nodule detection. Their algorithm learnt how to distinguish nodules from the initial surroundings and added difficult patches gradually. Maicas et al. (2018) used a teacher-student curriculum learning strategy for breast screening classification from DCE-MRI. They trained their model on simpler tasks before introducing the final problem of malignancy detection. Berger et al. (2018) proposed to use an adaptive sampling strategy to improve the segmentation performance on difficult regions in multi-organ CT segmentation.

### 2.4 Contributions

There are three major contributions of this work:

- To the authors’ knowledge, this is the first paper that provides a thorough analysis of machine learning methods for automatic cine CMR motion artefact detection on a large scale in-vivo database;
- A synthetic data augmentation strategy is proposed using k-space corruption to simulate motion artefact data (see Fig. 1c) of varying levels of severity;
- A curriculum learning strategy is employed using the synthetic data to efficiently train deep learning models with training samples of increasing difficulty.

This paper builds upon our previous work (Oksuz et al., 2018), in which we proposed the use of synthetically generated mistriggering artefacts in training a CNN. Here, we extend this idea to include both breathing and mistriggering artefacts and also use different levels of corruption to enable the curriculum learning strategy to be introduced.

## 3 Materials

We evaluate our approach using a subset of the UK Biobank data set. The UK Biobank CMR data were acquired using a common imaging protocol at one of a small number of study centres in the UK. The subset consists of short-axis cine CMR images of 3510 subjects. This subset was chosen to be free of other types of image quality issues such as missing axial slices and was visually verified by an expert cardiologist. The short-axis images have an in-plane image resolution of 1.8 × 1.8 mm^2^ with a slice thickness of 8.0 mm and a slice gap of 2 mm. A short-axis image stack typically consists of approximately 10 image slices and covers the full heart. The images’ matrix sizes vary from 120 to 280 pixels. Each cardiac cycle consists of 50 time frames and the full sequence of 50 balanced steady-state free precession (bSSFP) magnitude images were used for analysis. Details of the image acquisition protocol can be found in Petersen et al. (2015).

The data for the 3510 subjects consist of 3360 good quality acquisitions and 150 acquisitions with motion artefacts. The artefact acquisitions featured 57 mistriggering artefacts, 46 breathing artefacts, 42 arrythmia artefacts and 5 mixed artefacts. Binary image quality labels were generated by visual inspection and validated by an expert cardiologist.

## 4 Methods

In this section we first describe the neural network architectures used for motion artefact detection. We describe the preprocessing steps, then we detail the two network architectures used for motion artefact detection. We detail the data augmentation strategies to balance the classes and the curriculum learning setup proposed for training the network. Finally, we explain the details of the loss function and the optimisation of the networks.

### 4.1 Preprocessing

**Fig. 2:**
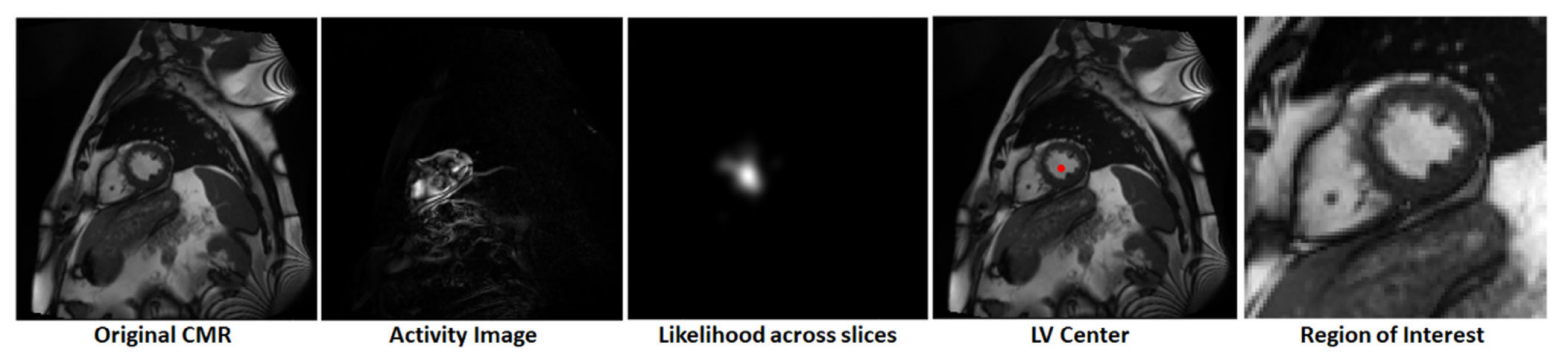
Region of interest extraction using Fourier transform in the temporal domain.

To circumvent problems related to different image resolutions and to enable efficient memory usage we use a region-of-interest (ROI) mechanism to extract regions of consistent size (illustrated in Fig. 2). Similar to Korshunova et al. (2016), we exploit the fact that each slice sequence captures one heart beat and use Fourier analysis to produce an image that captures the maximal activity at the corresponding heart beat frequency. From these activity images, we estimate the location of the centre of the left ventricle by combining the Hough circle transform with a custom kernel-based majority voting approach across all short axis slices. First, for each Fourier image (resulting from a single slice), the highest scoring Hough circles for a range of radii were found, and from all of those, the top 10 highest scoring ones were retained. Finally, a likelihood surface (centre image in Fig. 2) was obtained by combining the centres and scores of the selected circles for all slices. Each circle centre was used as the centre for a Gaussian kernel, which was scaled with the circle score, and all of these kernels were added. The maximum across this surface was selected as the centre of the ROI and 80 × 80 regions were extracted for further processing. The preprocessing strategy was able to correctly identify the heart region for all cases and was validated using the myocardial masks.

### 4.2 Deep learning models

We use deep learning methods that are capable of detecting temporal dependencies in a cine sequence. In this section, we detail the two different types of video classification methods namely; 3D CNN and LRCN.

**Fig. 3:**
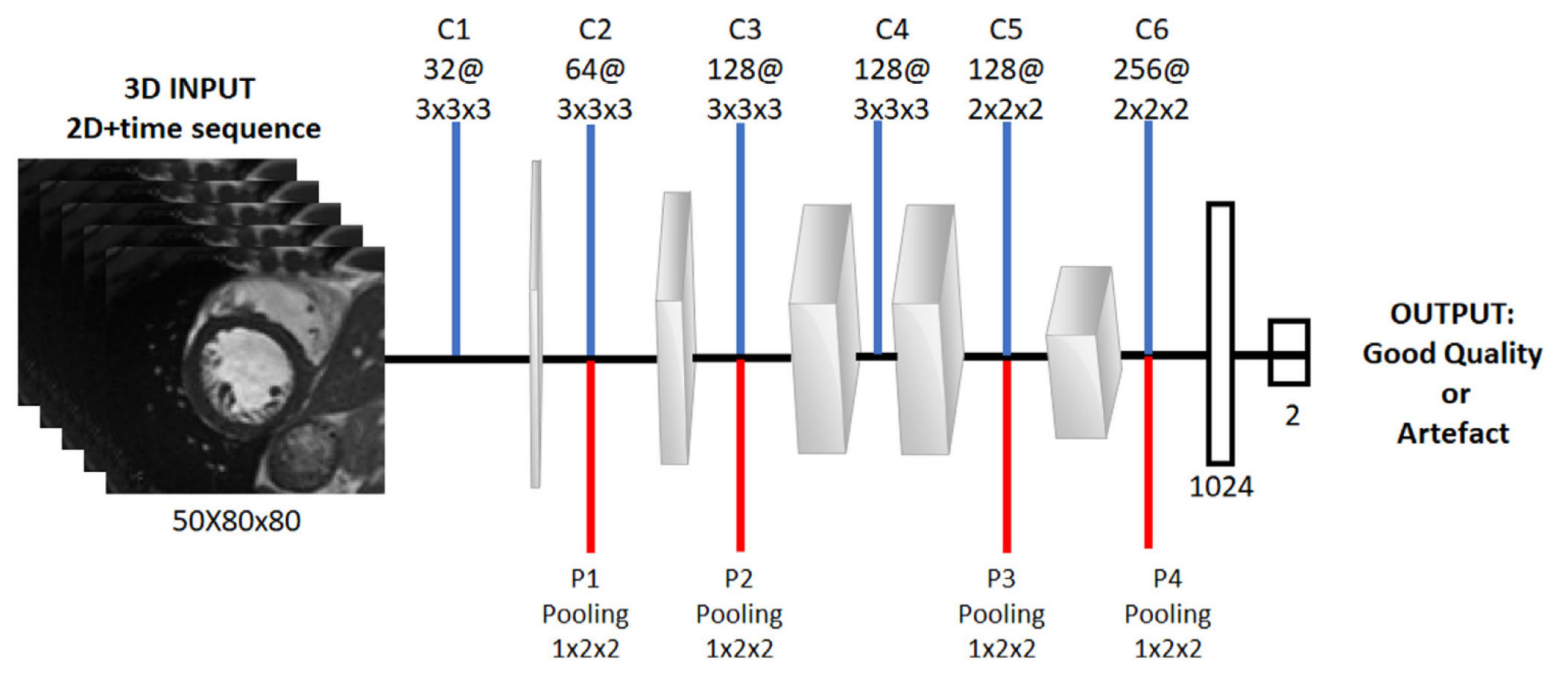
The 3-dimensional CNN architecture for motion artefact detection. Blue lines represent convolution operations and red lines correspond to the pooling operations following convolutional layers at each layer. The final two layers are densely connected layers of 1024 and 2 nodes respectively. (For interpretation of the references to colour in this figure legend, the reader is referred to the web version of this article.)

**3D CNN**: The proposed CNN consists of eight layers as visualised in Fig. 3. The architecture of our network follows a similar architecture to that proposed in Tran et al. (2015), which was originally developed for video classification using a spatio-temporal 3D CNN. In our case we use the third dimension as the time component for mid-ventricular sequences for classification. The input is an intensity normalised 80 × 80 cropped CMR image with 50 time frames as described in Section 4.1. The network has 6 convolutional layers and 4 pooling layers, 2 fully-connected layers and a softmax loss layer to predict motion artefact labels. After each convolutional layer a Rectifier Linear Unit (ReLU) activation is used. We then apply pooling on each feature map to reduce the filter responses to a lower dimension. We apply dropout with a probability of 0.5 at all convolutional layers and after the first fully connected layer to enforce regularisation. All of these convolutional layers are applied with appropriate padding (both spatial and temporal) and stride 1, thus there is no change in terms of size from the input to the output of these convolutional layers.

**Fig. 4:**
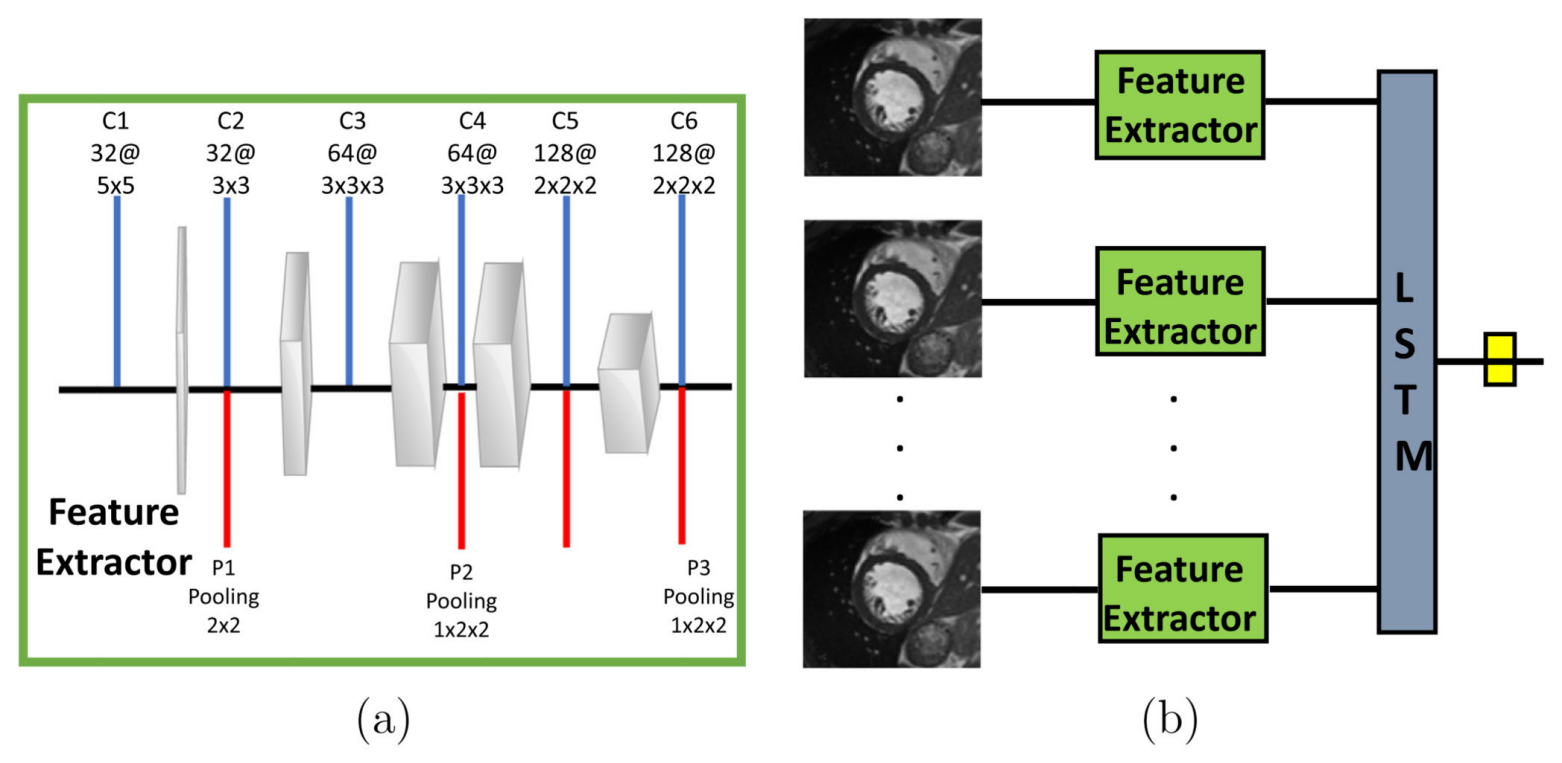
The LRCN architecture for motion artefact detection. (a) The feature extractor block for 2D images. Blue lines represent convolution operation and red lines correspond to the pooling operations following convolutional layers at each layer. (b) The network architecture. Multiple 2D inputs of different cardiac phases are passed through the feature extractor and a recurrent block (LSTM) is used for the final classification. (For interpretation of the references to colour in this figure legend, the reader is referred to the web version of this article.)

**LRCN:** The proposed Long-term Recurrent Convolutional Network model follows a similar strategy to that proposed in Donahue et al. (2017), which combines a deep hierarchical visual feature extractor (such as a CNN) with a model that can learn to recognise and synthesise temporal dynamics for tasks involving sequential data. The method works by passing each visual input *x_t_* (an image in isolation, or a frame from a video) through a feature transformation *ϕ* (usually a CNN), to produce a fixed-length vector representation. In our algorithmic setup, we use a feature extractor network to produce the feature representation and pass it to a LSTM unit to make the final prediction. Fig. 4 illustrates the architecture of our network. Our feature extractor block consists of 6 convolutional layers and 3 pooling layers and vectorises the final output to be used in a recurrent fashion.

### 4.3 Balancing the classes

In order to address the heavy class imbalance in our data set we propose to generate synthetic artefacts using knowledge of the cine MR acquisition process. Cine CMR images are acquired using ECG triggering and typically the full k-space of one image is filled over multiple beats during a breath hold. During the acquisition mistakes with ECG-triggering can cause k-space lines to be filled with data from an incorrect cardiac phase. Similarly, breathing motion of the patient can cause k-space lines to be filled with data from a different anatomical location. We aim to simulate these mistriggering and breathing artefacts at varying levels of severity to be able to utilise a curriculum learning strategy during training.

#### 4.3.1 Mistriggering artefacts

**Fig. 5:**
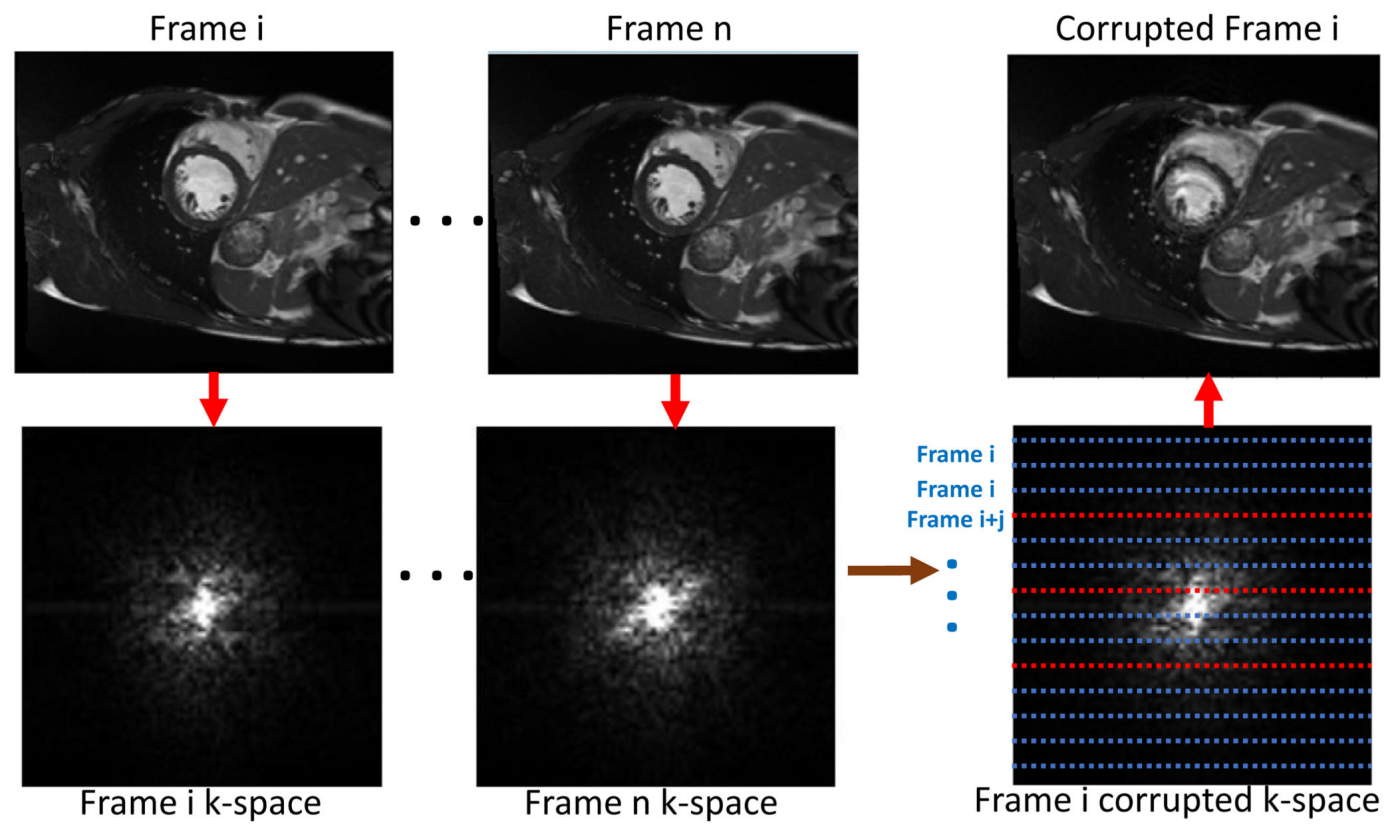
K-space corruption for mistriggering artefact generation in k-space. The Fourier transform of each image frame is applied to generate the k-space representation of each image. We replace k-space lines with lines from different temporal frames to generate corruptions.

The UK Biobank data set that we use was acquired using Cartesian sampling and we follow a Cartesian k-space corruption strategy to generate synthetic but realistic motion artefacts. We first transform each 2D short axis sequence to the Fourier domain and change 1 in *z* Cartesian sampling lines to the corresponding lines from other cardiac phases in order to mimic cardiac motion artefacts. By using different values for *z*, we are able to generate cardiac motion artefacts with different severity. In Fig. 5 we show an example of the generation of a corrupted frame *i* from the original frame *i* using information from the k-space data of other temporal frames. We add a random frame offset *j* when replacing the lines.

Using this approach, the original good quality images from the training set are used to increase the total number of low quality images in the training set. This is a realistic approach as the motion artefacts that occur from mistriggering arise from similar misallocations of k-space lines.

#### 4.3.2 Breathing artefacts

Following a similar idea to Lorch et al. (2017) we produce breathing artefacts by applying 2D translations to the image frames prior to generating their k-space representations. The translations follow a sinusoidal pattern. To simulate a subject that completed four breathing cycles within one acquisition with 256 phase-encoding steps, we sampled a sinusoidal curve with four cycles at 256 time points to produce the translations. Once the k-space representations of the frames were generated in this way they were combined in the normal way and reconstructed into images.

**Fig. 6:**
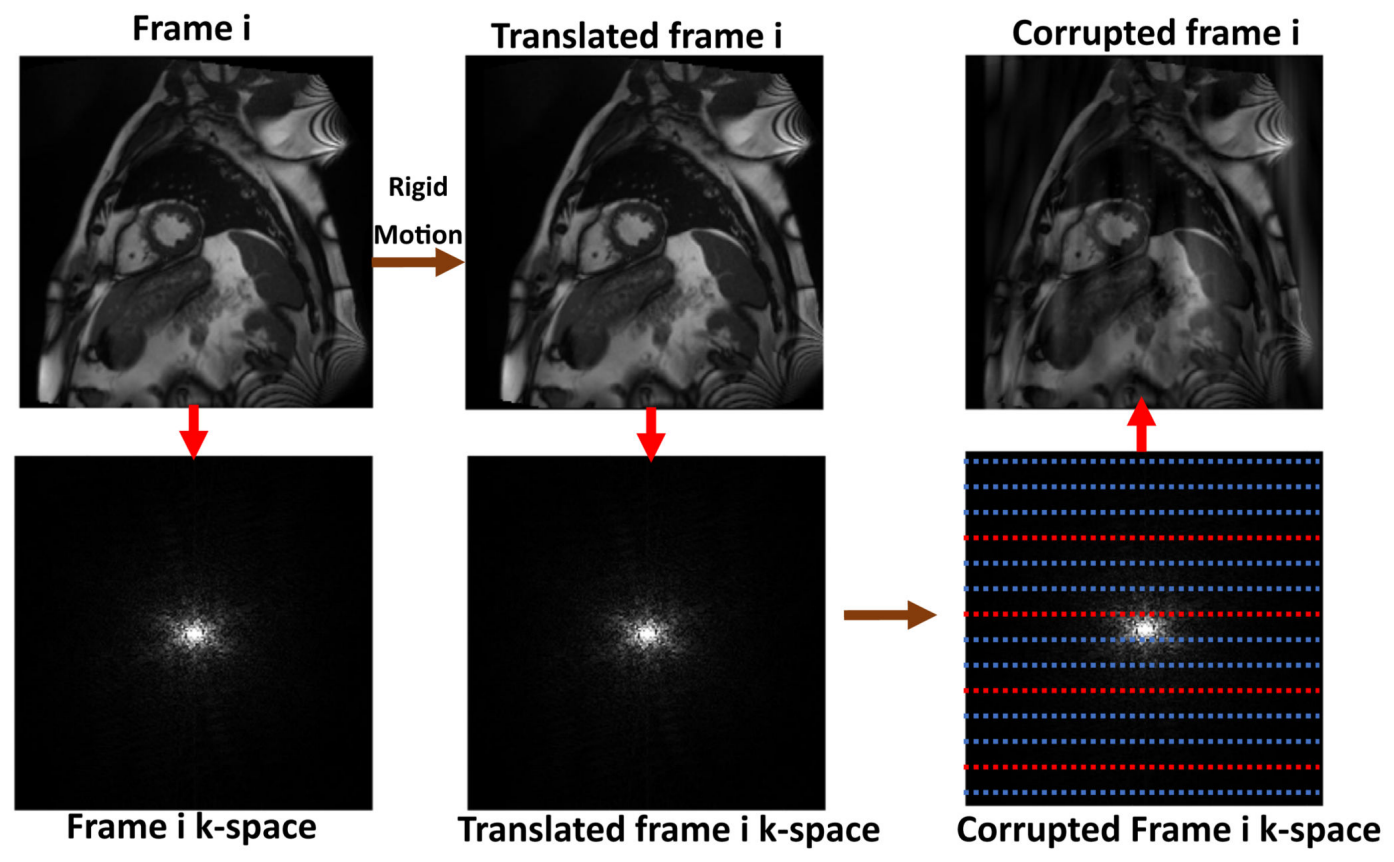
K-space corruption for breathing artefact generation in k-space. The Fourier transform is applied to generate the k-space of each image frame and we replace k-space lines with lines from frames with different 1D translations which follow a sinusoidal pattern to simulate repository motion.

In Fig. 6 we show an example of the generation of a corrupted frame *i* from the original frame *i* using information from the k-space data of other translated frames^1^ (Cruz et al., 2016).

### 4.4 Curriculum learning

We propose to use baby-step^2^ curriculum learning during training of the networks to leverage the additional data resulting from the k-space corruption strategy. We start the network training with heavily corrupted images (easy examples) and gradually introduce less corrupted images (hard examples).

Formally, we have a training data set of images *D* = (*I*_1_*, y*_1_*), . . . , (I_n_, y_n_),* where *I_i_* ∈ *R^d^* denotes the *i*th cardiac sequence of training samples, *y_i_* represents its label and *n* is the number of training samples. The estimated label ${\hat{y}}_{i}$ is predicted by *f*(*I_i_, W*), where *W* represents the model parameters of the decision function *f*. Let *L*(*y_i_, f*(*I_i_, W*)) denote the loss function which calculates the cost between the ground truth label *y_i_* and the estimated label ${\hat{y}}_{i}=f\left(x_{i},W\right).$ The motion artefact detection is then optimised by:

$$W^{*}=\underset{W}{\text{argmin}}\sum_{i=1}^{n}L\left(y_{i},f\left(I_{i},W\right)\right).$$

**Fig. 7:**
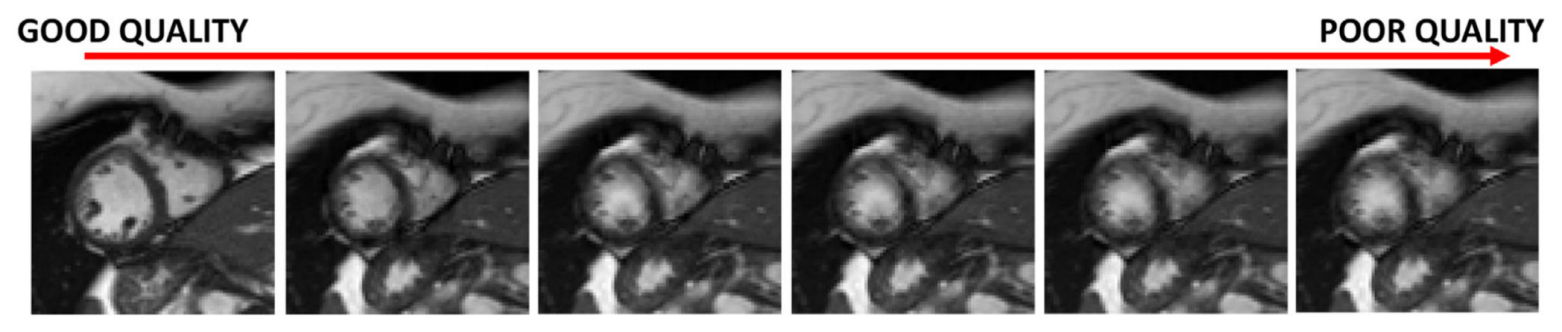
Gradual corruption using mistriggering type synthetic artefact generation for curriculum learning. The myocardial borders and papillary muscles become more blurred with the severity of the artefacts and it is harder to distinguish those structures under severe artefact cases.

**Fig. 8:**
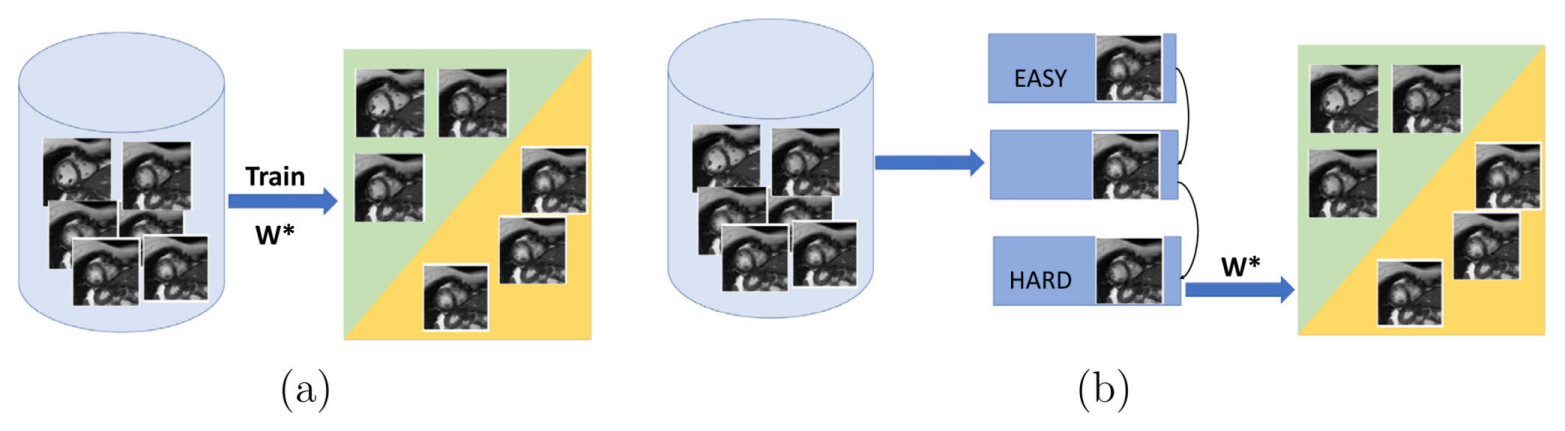
Curriculum learning using motion artefacts generated with various levels of severity. (a) The traditional way to train a model fails to consider the complexity of image quality detection where introducing noisy or difficult samples early in training may impair model performance. (b) The training data is divided into different difficulty levels based on a predetermined curriculum. The training procedure progresses from easy to hard image samples, which guides the model to achieve better performance. (The illustration of complexity is shown in Fig. 7).

Here, W* denotes the optimal model parameters. We utilise the different levels of corruption achieved by the k-space corruption strategy (as visualised in Fig. 7) to sort the training samples according to their difficulty for classification. This leads to the proposed algorithm illustrated in Fig. 8. We first group image sequences into subsets based on the corruption level of the poor quality image (i.e., from high level of corruption to low level of corruption). We then train the model via iterative learning using increasingly corrupted images as described in Algorithm 1.The notation *D^i^* is defined in the input of the algorithm, i.e. i indicates the number of the training set in the curriculum, and there are *b* training sets in total. The clustering into subgroups according to artefact severity is only done for the synthetic images, and we introduce them only gradually in the training. The original artefact cases from the dataset are used at every stage of the curriculum learning, since we do not have any information regarding the severity of these artefacts.

**Algorithm 1: Proposed curriculum learning strategy for motion artefact detection.**

**INPUT:** Data set of synthetically generated image sequences $D={\left\{D^{i}\right\}}_{i=1}^{b}$ ordered by a pre-defined curriculum

**OUTPUT:** Optimized model parameters W*

1:  *D*^train^ = Original Data set of Image Sequences

2:  **for** i={1,…,b} **do**

3:        *D*^train^ = *D*^train^ ∪ *D^i^*

4:        **for** epoch={1,…,k} **do**

5:             train (*W*, *D^train^*)

6:        **end for**

7:        select best W*

8:  **end for**

### 4.5 Loss functions and optimisation

The training of a CNN can be viewed as a combination of two components: a loss function or training objective, and an optimisation algorithm that minimises this function. In this study, we use the stochastic gradient descent (SGD) optimiser to minimise the binary cross entropy. The cross entropy represents the dissimilarity of the approximated output distribution from the true distribution of labels after a softmax layer and is defined as:

$$L=\frac{-1}{n}\sum_{i=1}^{n}\left[y_{i}\;log\left({\hat{y}}_{i}\right)+\left(1-y_{i}\right)log\left(1-{\hat{y}}_{i}\right)\right].$$

The training converges when the network does not significantly improve its performance on the validation set for a predefined number of epochs (100). An improvement is considered sufficient if the relative increase in performance is at least 0.5%.

During training of LRCN and CNN, a batch-size of 50 2D+time sequences was used due to memory constraints. The momentum of the optimiser was set to 0.90 and the learning rate was 0.0001. The parameters of the convolutional and fully-connected layers were initialised from a zero mean, unit standard deviation Gaussian distribution. In each trial, training was continued until the network converged. Convergence was defined as a state in which no substantial progress was observed in the training loss. Parameters were optimised using a grid-search among all parameters. We used the Keras Framework with Tensorflow backend for implementation and training the network with the curriculum learning setup took around 12 hours on a NVIDIA Quadro 6000P GPU. Classification of a single 2D+time image sequence took less than 1s.

## 5 Experiments and results

Three sets of experiments were performed. The first set of experiments (Section 5.2) aimed to compare the performance of the different algorithmic approaches for automatic motion artefact detection, while the second set of experiments (Section 5.3) aimed at comparing different design choices for balancing the classes. In Section 5.4 we validate the proposed curriculum learning training strategies. Finally, we visualize saliency maps in Section 5.5 and evaluate our algorithm on multi-class artefact detection task in Section 5.6. All experiments were carried out using the Python programming language, using standard Python libraries, Tensorflow, Keras and the scikit-learn Python toolkit (Pedregosa et al., 2011). Before describing the experiments in detail, we first describe the evaluation measures used.

### 5.1 Evaluation metrics and methods of comparison

A 10-fold repeated stratified cross-validation was used to validate the performance of each algorithm. In each fold, the classification accuracy (i.e. the proportion of subjects correctly classified), as well as the recall (the proportion of artefact images correctly classified) and the precision (the proportion of correctly classified good quality images) were computed. Finally, we computed the average balanced accuracies and the area under the ROC curve (AUC). The accuracy, precision, recall, balanced accuracy and AUC metrics are defined as:

$$\text{Accuracy}=\frac{TP+TN}{TP+FP+FN+TN},$$

$$\text{Precision}=\frac{TP}{TP+FP}\;\text{Recall}=\frac{TP}{TP+FP},$$

$$\text{Balanced}\;\text{Accurancy}=\frac{\text{Precision}+\text{Recall}}{2},$$

$$\text{AUC}=\int_{-\infty }^{\infty }\text{TPR}(t)\text{FPR}(t)dt.$$

where *TP* represents true positives, *FP* is false positives, *FN* is false negatives and *TN* is true negatives. TPR defines the true positive rate and FPR defines the false positive rate for a given threshold *t*.

We compared our algorithm with a range of alternative classification techniques: K-nearest neighbours, Support Vector Machines (SVMs), Decision Trees, Random Forests, Adaboost and Naive Bayesian. The inputs to all algorithms were the cropped intensity-normalised data as described in Section 4.1 with the exception of the method proposed by Lorch et al. (2017). For this method, we used hand crafted features (e.g. box, line, texture and histogram) to train a decision forest algorithm similar to Lorch et al. (2017). We optimised the parameters of each comparative algorithm using a grid search. We also tested two techniques developed for image quality assessment in the computer vision literature: the NIQE metric (Mittal et al., 2013) is based on natural scene statistics and was trained using a separate set of 50,000 2D good quality CMR images to establish a baseline for good image quality; and the Variance of Laplacians is a moving filter that has been used to detect the blur level of an image. For both of these techniques we used a 10-fold SVM for classification of the final scores.

### 5.2 Synthetic data

We first tested our algorithm using synthetically generated artefacts to evaluate its performance. We generated different levels of corruption from good quality images using the pipeline explained in Section 4.3 and evaluated the algorithms on a balanced data set consisting of 3360 good quality and 3360 artefact images with different severity. We used a 10-fold cross validation to classify the good quality and artefact images. The results are reported in Tables 1 and 2 for breathing and mistriggering artefacts respectively. The high performance of the deep learning architectures is evident for both types of artefact. LRCN and 3D-CNN show the highest performance in terms of accuracy, recall and balanced accuracy. The general high performance by all methods can be explained by the low complexity of the problem (i.e. original vs. synthetically corrupted version of the same image) and the availability of the balanced data set.

### 5.3 Augmentation technique analysis

We evaluated deep learning algorithms on the real in-vivo cases using 150 artefact and 3360 good quality images. We tested six different training configurations of two neural network strategies to evaluate their performance in more detail: (1) training using only acquired magnitude data without any data augmentation, (2) training using translational data augmentation with translations only, (3) training using Gaussian blurring corrupted data augmentation, (4) training using data augmentation with mistriggering k-space corrupted data only, (5) training using data augmentation with breathing k-space corrupted data only, (6) training using both mis-triggering and breathing type synthetic artefacts, (7) cost-sensitive learning with a weighted cost function. The cost sensitive learning used a weighted binary cross entropy loss function with the weights determined by the ratio of samples in the classes (150: 3360). We also augmented data in this setup using translations for a fair comparison, but the data augmentation was not used to balance the classes in this scenario and the augmentation was applied in the same way for both classes. Moreover, we have used Gaussian blurring to corrupt the data at different levels to showcase the performance of an additional data corruption strategy. In each setup, the acquired data corrupted by motion artefacts were used together with the real motion artefact data.

The translational data augmentation used random shifts in both the horizontal and vertical directions in the range of [W/5, H/5], where W and H represent the width and height of the image respectively (i.e. W=H=80 pixels=144 mm in our case). Rotations were not used due to their influence on image quality caused by the necessary interpolation. Note that none of the augmented data were used for testing. They were only used for increasing the total number of training images.

We used a 10-fold stratified cross validation strategy to test all algorithms, in which each image appeared once in the test set over all folds. Due to the high class imbalance, all algorithms achieved over 0.9 accuracy and so we do not report this metric in Table 3. The interesting comparison for the methods lies in the recall numbers, which quantify the capability of the methods to identify images with artefacts. The results show that the LRCN-based technique is capable of identifying the presence of motion artefacts with high recall compared to the other techniques.

### 5.4 Influence of curriculum learning

We investigated the influence of curriculum learning on our algorithm. For these experiments we used the best performing model from Table 3, namely the LRCN model with a mixture of breathing and mistriggering synthetic artefacts. During generation of the synthetic training samples we used *b* = 10 different levels of k-space corruption and used these to generate the curriculum. We introduced the easy samples (highly corrupted images) at the beginning of the training and gradually included harder samples (less corrupted images).

**Fig. 9:**
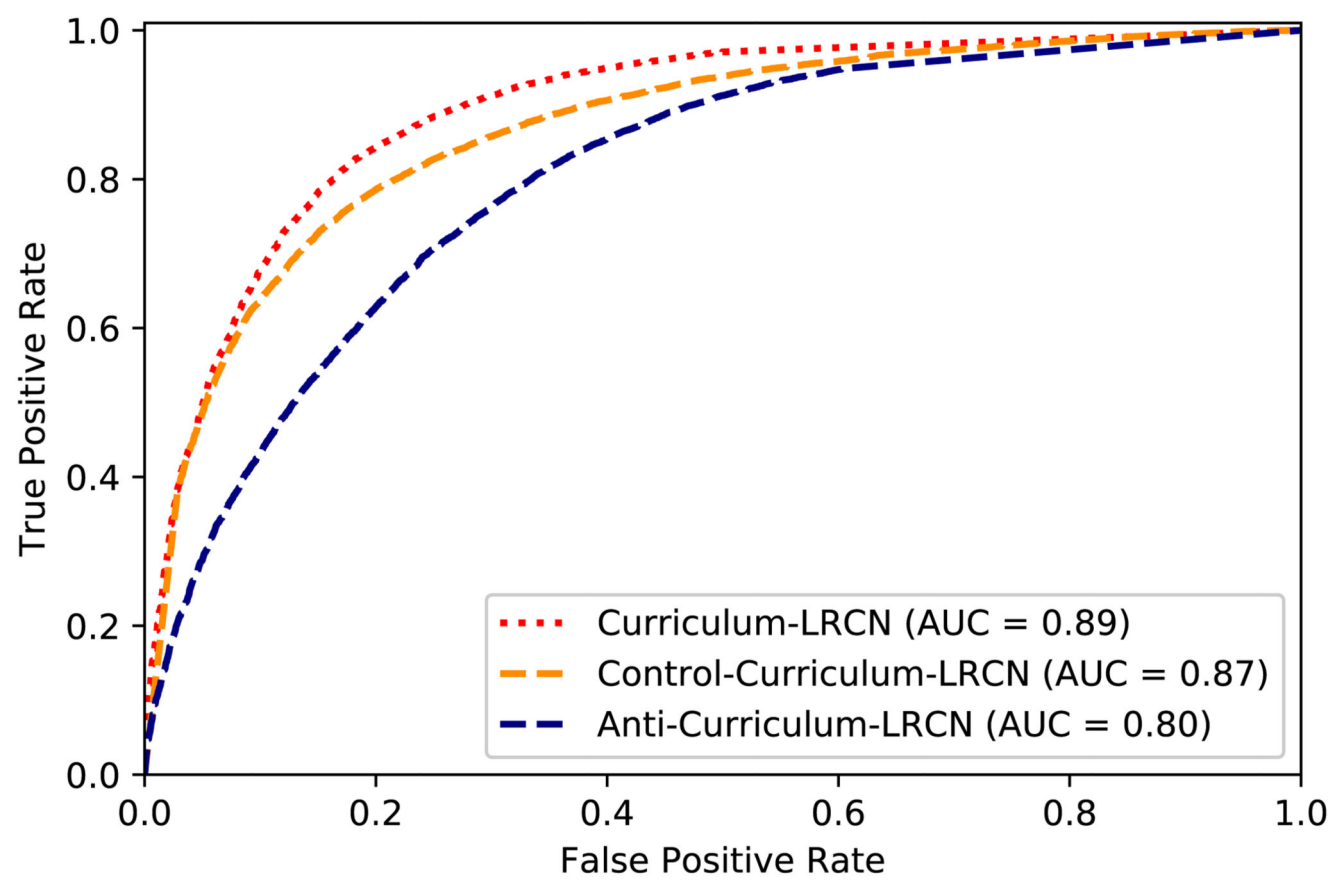
ROC curves for the LRCN-based motion artefact detection approach using curriculum learning. Gradually introducing harder samples during training improves the performance of the algorithm compared to the random (control-curriculum) and harder-to-easier configurations (anti-curriculum).

In order to evaluate the success of this approach, we compared to two alternatives. First, we repeated the curriculum generation process in the opposite way and first used hard samples and gradually introduced easier samples (anti-curriculum). Second, we used a curriculum consisting of a random set of samples with no sorting at each run (control-curriculum). Fig. 9 shows ROC curves and reports AUC values for these three approaches. We performed a Delong’s statistical significance test (DeLong et al., 1988) to evaluate the differences between the methods. The curriculum learning strategy significantly outperformed random sampling and anti-curriculum learning (*p*-value < 0.05).

**Fig. 10:**
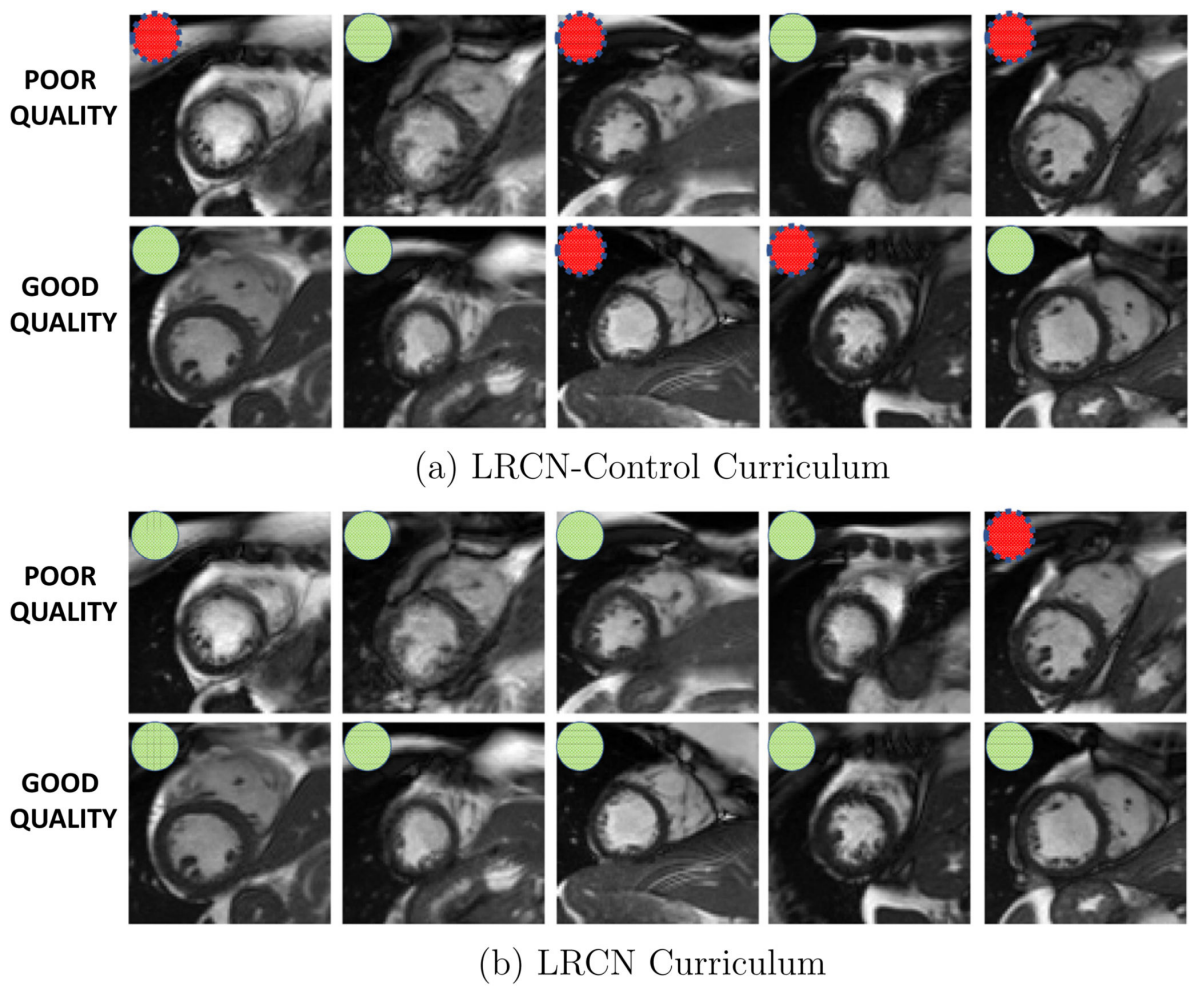
Curriculum learning improves the classification of motion artefacts on borderline cases. (a) shows the results of control-curriculum with coloured circles for good and poor quality images. (b) illustrates the results of the curriculum learning strategy for the same samples. Most of the borderline cases are correctly identified with the curriculum learning strategy. The green circles indicate the correct classifications and red circles indicate the wrong classifications by the methods. (For interpretation of the references to colour in this figure legend, the reader is referred to the web version of this article.)

We show the improvements in classification using curriculum learning using samples from the data set in Fig. 10. Some difficult classification cases were selected to showcase the performance of both methods. The figure shows the borderline cases from both classes, where there is only a slight difference between the good and poor quality images. The use of curriculum learning enables detection of borderline cases of motion artefacts (poor quality images) with great success compared to control-curriculum.

### 5.5 Saliency maps

**Fig. 11:**
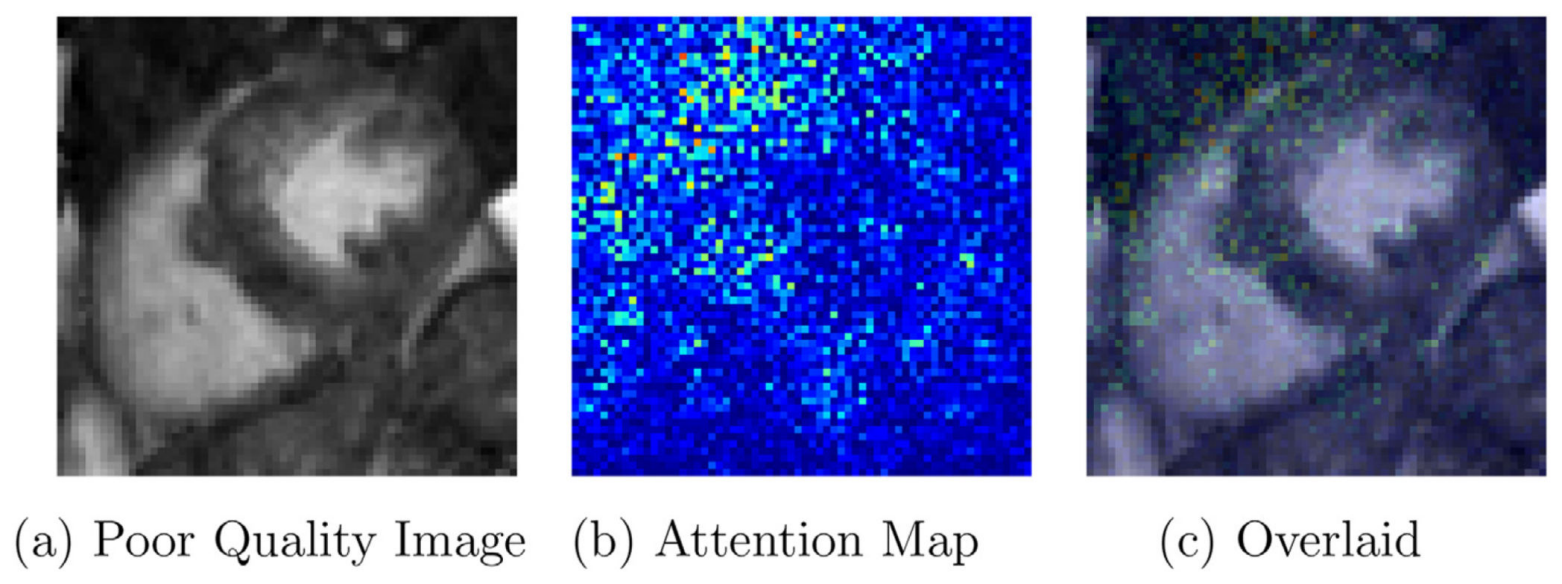
Attention map for a poor quality image learned by the last layer of the network, where red indicates high attention and blue low attention. The network architecture captures the area of significance for correctly classifying the image. Results provided for LRCN network trained with curriculum learning. (For interpretation of the references to colour in this figure legend, the reader is referred to the web version of this article.)

Attention map (Simonyan et al., 2013), uses the gradients of any target concept (e.g. logits), flowing into the final convolutional layer to produce a coarse localization map highlighting the important regions in the image for predicting the concept. To visualize activation over final dense layer outputs, we need to switch the softmax activation out for linear since gradient of output node will depend on all the other node activations. Fig. 11 shows the attention maps of the last layer of the network on an example from the test set. Attention maps provide a way to visualize the most influential areas in the input data used for the classification. In poor quality images the activations are high in blurry regions as visualized in the Fig. 11.

### 5.6 Multi-class detection

We test our LRCN algorithm with curriculum learning on a multi-class classification task to evaluate the capability of our model on classifying between breathing vs. gating-related artefacts. We used breathing k-space corruption and mis-triggering k-space corruption respectively at each curricula to balance the classes similar to previous experiments. In Table 4, we report the balanced accuracy results for two state of the art technique results to show the improved performance with our method. As expected with fewer number of cases in the dataset the classification task is more difficult. The results indicate the potential of our method in class-specific artefacts, which can be instrumental in addressing the image quality issues.

## 6 Discussion and conclusion

We have presented an extensive study on automatic cardiac motion artefact detection using spatio-temporal deep learning techniques. The motion artefact detection problem exhibits a severe data imbalance between the classes in UKBB dataset. Our fundamental contribution in this paper is to address this data imbalance by using a k-space based corruption strategy to increase the robustness of the classification. With a variety of synthetic data generation techniques we propose to augment data for training the classifier using knowledge of the cine MR acquisition process. We have also investigated the robustness of two deep learning architectures developed for video classification for classifying motion-related artefacts. Benefiting from the controlled environment of synthetic data generation we utilised curriculum learning for training and showcased the efficiency of the technique in comparison with other data sampling strategies.

One key observation of our work is the superiority of deep learning methods to classify motion artefacts compared to other state-of-the-art machine learning algorithms. Moreover, we tested data augmentation strategies extensively and illustrated the superior performance of k-space corruption to generate synthetic data for augmentation. It is interesting to observe that using different corruption strategies improves the performance of the classification techniques. Finally, employing a curriculum learning strategy for training the image classification networks ensured better performance compared to anti-curriculum and random sampling strategies.

In the future, we would like to investigate novel loss functions for the detection of image quality. Moreover, investigation of basal and apical slice quality, which exhibits a slightly different anatomy and challenge, is an important future direction. In this work, we deliberately used existing network architectures and loss functions to enable us to focus our evaluation on the influence of our novel data augmentation and curriculum learning strategies. In future work we would like to investigate novel architectures tailored to the problem at hand. Moreover, a regression based approach on artefact detection could evaluate the impact of artefacts on downstream tasks (e.g. how badly would the artefacts affect segmentation accuracy or calculation of metrics such as ejection fraction).

The UK Biobank is a controlled study and the number of images with motion artefacts is limited. In real clinical acquisitions the likelihood of motion artefact occurrence is higher (although the classes would still be imbalanced), and there would be great value for motion artefact detection mechanisms being deployed ‘on-the-fly’ in the MR scanner. The indication for CMR is often prognostic stratification of already existent cardiac diseases and patients are more likely to have arrythmias, have difficulties with breath-holding or remaining still during acquisition. With the successful translation of such tools in clinical setups high diagnostic image quality could be ensured on the spot. Indeed, these mechanisms would not necessarily need to be CMR specific and could even be applied to different medical image modalities and different organs. With this aim in mind, we would like to validate our algorithm on multi-vendor and multi-site artefact datasets in the future.

In conclusion, we believe that the work that we have presented represents an important contribution to the understanding of CMR image quality assessment. Our novel ideas of leveraging k-space corruption for data augmentation and training the classifier with a curriculum learning strategy have been shown to improve motion artefact detection accuracy. In the current environment of the increasing use of imaging in clinical practice, as well as the emergence of large population data cohorts which include imaging, our proposed automated quality control methods can ensure the accuracy of subsequent analysis pipelines.

## Supplementary Material

Supplementary material associated with this article can be found, in the online version, at doi:10.1016/j.media.2019.04.009.

Supplementary Data S3

Supplementary Data S2

Supplementary Data S1

## Figures and Tables

**Table 1:** Synthetic mistriggering artefact data classification results for mean accuracy (A), precision (P), recall (R) and balanced accuracy (BA) results. A 10-fold cross validation was used and each image was labelled once over all folds and standard deviation over folds is reported (mean ± std). All results are multiplied by 1000 and the bold font highlights the best results.

Methods	A	P	R	BA
K-Nearest Neighbours	742 ± 25	742 ± 33	746 ± 40	744 ± 37
Linear SVM	748 ± 36	743 ± 89	749 ± 41	746 ± 73
Decision Tree	756 ± 42	757 ± 46	751 ± 33	754 ± 41
Random Forests	787 ± 45	782 ± 78	786 ± 62	784 ± 67
Adaboost	783 ± 37	781 ± 60	778 ± 73	779 ± 66
Naive Bayesian	809 ± 65	796 ± 48	804 ± 57	800 ± 52
Variance of Laplacian	802 ± 42	799 ± 62	803 ± 79	802 ± 41
NIQE	922 ± 56	919 ± 72	925 ± 82	923 ± 71
Lorch et al. (2017)	893 ± 62	892 ± 83	894 ± 49	893 ± 22
**3D CNN**	961 ± 79	957 ± 101	959 ± 87	958 ± 74
**LRCN**	**963 ± 45**	**963 ± 33**	**965 ± 41**	**964 ± 38**

**Table 2:** Synthetic breathing artefact data classification results for mean accuracy (A), precision (P), recall (R) and balanced accuracy (BA) results. A 10-fold cross validation was used and each image was labelled once over all folds and standard deviation over folds is reported (mean ± std). All results are multiplied by 1000 and the bold font highlights the best results.

Methods	A	P	R	BA
K-Nearest Neighbours	718 ± 33	724 ± 37	721 ± 30	723 ± 36
Linear SVM	740 ± 41	737 ± 80	744 ± 48	741 ± 76
Decision Tree	707 ± 55	708 ± 42	713 ± 37	711 ± 48
Random Forests	764 ± 56	776 ± 64	781 ± 68	778 ± 61
Adaboost	768 ± 39	768 ± 54	772 ± 50	770 ± 57
Naive Bayesian	788 ± 70	790 ± 43	797 ± 68	793 ± 42
Variance of Laplacian	809 ± 43	820 ± 69	824 ± 55	822 ± 59
NIQE	897 ± 59	899 ± 71	904 ± 61	902 ± 50
Lorch et al. (2017)	896 ± 62	895 ± 47	897 ± 38	896 ± 77
**3D CNN**	953 ± 89	951 ± 91	961 ± 82	955 ± 70
**LRCN**	**961 ± 41**	**962 ± 29**	**964 ± 51**	**963 ± 30**

**Table 3:** Mean balanced accuracy (BA), precision (P), recall (R) and area under the ROC curve (AUC) results of image classification for motion artefacts (in-vivo data set) trained on real and synthetic data sets. A 10-fold cross validation was used and each image was labelled once over all folds(mean ± std). t-aug,g-aug, m-aug, b-aug represent translational, gaussian blurring, mistriggering and breathing type augmentations respectively. b-m-aug represents a random mix of mis-tiggering and breathing artefacts to balance the data set. cs stands for cost-sensitive learning with weighted losses. All results are multiplied by 1000 and the bold font highlights the best results.

Methods	BA	P	R	AUC
3DCNN no-aug	590 ± 85	713 ± 69	467 ± 82	581 ± 124
3DCNN t-aug	679 ± 63	751 ± 54	607 ± 78	674 ± 87
3DCNN g-aug	690 ± 69	709 ± 101	670 ± 91	685 ± 90
3DCNN m-aug	717 ± 71	762 ± 78	673 ± 74	732 ± 71
3DCNN b-aug	695 ± 62	703 ± 40	687 ± 98	699 ± 67
3DCNN cs	515 ± 91	503 ± 57	520 ± 68	613 ± 50
3DCNN b-m-aug	721 ± 47	**768 ± 61**	673 ± 40	735 ± 67
LRCN no-aug	629 ± 97	724 ± 57	533 ± 65	603 ± 71
LRCN t-aug	664 ± 55	722 ± 69	607 ± 87	704 ± 73
LRCN g-aug	698 ± 61	715 ± 73	672 ± 80	708 ± 84
LRCN m-aug	731 ± 77	743 ± 77	720 ± 128	826 ± 80
LRCN b-aug	719 ± 53	731 ± 81	707 ± 81	759 ± 93
LRCN cs	511 ± 89	502 ± 72	520 ± 48	608 ± 71
**LRCN b-m-aug**	**74 ± 50**	751 ± 84	**733 ± 66**	**828 ± 57**

**Table 4:** Multi-class detection of motion artefact. The balanced accuracy results of 3-class classification (Good quality, breathing and triggering-based). A 10-fold cross validation was used and each image was labelled once over all folds and standard deviation over folds is reported. All results are multiplied by 1000 and the bold font highlights the best results.

Methods	Breathing	Mis-triggering and Arrythmia
Random Forests	658 ± 129	689 ± 136
Variance of Laplacian	672 ± 118	687 ± 127
LRCN	710 ± 122	731 ± 126
**LRCN-Curriculum**	**741 ± 12**	**752 ± 114**

## Footnotes

1 Note that the original images themselves sometimes contain small artefacts such as the Moire artefacts seen in Fig. 6. However, these are normally far from the region of interest and so do not affect our approach.

2 The term baby-step refers to keeping the previously introduced training samples in the pool of training examples rather than replacing them with new ones.
